# Mei-P26 Cooperates with Bam, Bgcn and Sxl to Promote Early Germline Development in the *Drosophila* Ovary

**DOI:** 10.1371/journal.pone.0058301

**Published:** 2013-03-20

**Authors:** Yun Li, Qiao Zhang, Arnaldo Carreira-Rosario, Jean Z. Maines, Dennis M. McKearin, Michael Buszczak

**Affiliations:** 1 Department of Molecular Biology, University of Texas Southwestern Medical Center at Dallas, Dallas, Texas, United States of America; 2 Howard Hughes Medical Institute, Chevy Chase, Maryland, United States of America; National Cancer Institute, United States of America

## Abstract

In the *Drosophila* female germline, spatially and temporally specific translation of mRNAs governs both stem cell maintenance and the differentiation of their progeny. However, the mechanisms that control and coordinate different modes of translational repression within this lineage remain incompletely understood. Here we present data showing that Mei-P26 associates with Bam, Bgcn and Sxl and *nanos* mRNA during early cyst development, suggesting that this protein helps to repress the translation of *nanos* mRNA. Together with recently published studies, these data suggest that Mei-P26 mediates both GSC self-renewal and germline differentiation through distinct modes of translational repression depending on the presence of Bam.

## Introduction

*Drosophila* early germline development has served as a useful model for studying the transcriptional and translational hierarchies that control the maintenance of stem cells and the differentiation of their daughters [1]. Each *Drosophila* germarium contains two to three germline stem cells (GSCs) that lie immediately adjacent to a niche comprised of terminal filament and cap cells. Decapentaplegic (Dpp) produced by the cap cells activates a Bone Morphogenetic Protein (BMP) signal transduction cascade in GSCs that results in the transcriptional silencing of the differentiation factor *bag of marbles* (*bam*) [2], [3], [4], [5]. GSC daughters displaced from the niche express *bam* and undergo four incomplete mitotic divisions to form a 16-cell cyst.

In addition to the BMP pathway transcriptional based self-renewal mechanism, the Nanos-Pumilio complex and the miRNA pathway also help to promote stem cell maintenance, presumably through the direct translational repression of specific mRNAs that promote differentiation [6], [7]. Mutations in *nanos*, *pumilio* or several components of the miRNA pathway result in a rapid loss of GSCs [8], [9], [10]. Identifying the direct targets of these pathways specifically in GSCs remains technically difficult, but the Nanos-Pumilio and miRNA pathways likely target a variety of mRNAs involved in different aspects of the cell cycle and germline function [6], [7], [8], [9], [10].

Bam plays a central role in promoting the early differentiation of *Drosophila* germline stem cell daughters once they leave the cap cell niche [11], [12]. While the molecular function of Bam protein remains enigmatic, recent results have begun to suggest that Bam plays a role in regulating the translation of specific messages. Bam binds to Bgcn, a RNA helicase-like molecule, and represses Nanos expression in developing cysts through elements within the 3′UTR of *nanos* mRNA [13]. This repression appears functionally significant, as mis-expression of *bam* in ovaries expressing a *nanos*-*tubulin* 3′UTR transgene does not cause premature GSC differentiation [13].

Bam has also been found to either cooperate or act in parallel with the Sex-Lethal (Sxl) protein [14]. Sxl establishes the sexual identity of female cells by regulating sex-specific alternative splicing and translational repression [15]. Loss of *sxl*, or *sans-fille* (*snf*), a splicing factor needed for the proper germline expression of *sxl* [14], results in a tumor phenotype marked by the accumulation of multicellular germline cysts. These cells exhibit inappropriate expression of male specific transcripts in the female germline [14], [16], [17], [18], [19]. Interestingly, *bam* mutants also exhibit a similar mis-expression of male transcripts in the female germline, suggesting a functional link between Bam and Sxl [14]. A recent study shows that Sxl negatively regulates Nanos translation directly through specific binding sites in the 3′UTR of *nanos* transcripts [20]. Bam also appears to contribute to the control of *nanos* mRNA translation [13], but whether Bam directly contacts this target mRNA remains unclear. Genetic experiments provide further evidence that Sxl and Bam cooperate to repress nanos expression during the early steps of germline cyst development [20].

The TRIM-NHL domain protein Mei-P26 is another key regulator of germline differentiation. Mutations in *mei-P26* result in tumor formation in the ovary and testis, characterized by the accumulation of undifferentiated multicellular germline cysts, indicating that the protein plays an important role in germline differentiation [21], [22]. In addition, loss of *mei-P26* compromises BMP signal transduction within GSCs, suggesting that Mei-P26 regulates both stem cell maintenance and stem cell daughter differentiation [23]. How Mei-P26 switches between these distinct roles remains unclear. Interestingly, Neumuller et al. (2008) provided genetic evidence that *bam* needs *mei-P26* to promote the differentiation of germline cells. Whether this requirement is direct or indirect remains uncertain, but these findings suggest that Bam may act as a critical switch in regards to Mei-P26 function.

Using a series of biochemical and genetic experiments, we provide further evidence that Mei-P26 acts with Bam, Bgcn and Sxl to regulate the differentiation of stem cell daughters displaced away from the niche. These results highlight the importance of the function of a TRIM-NHL domain protein in controlling two distinct cell fates within the germline.

## Materials and Methods

### ***Drosophila*** Strains

*mei-p26^mfs1^* was a gift from R. S. Hawley [22]. *w^1118^*; *bam*P-Bam::HA/CyO; *bgcn*P-*bgcn*::GFP/TM3 and *hs-*Bam::HA transgenic lines have been described previously [13]. *y^1^ w^1118^ snf^148^ P{ry[+t7.2] = neoFRT}19A/FM6* (BL#:7398) was obtained from the Bloomington Stock Center.

### Immunohistochemistry

Ovaries were processed and imaged as previously described [23]. The following antibodies were used: rabbit anti-GFP (1 500; Invitrogen), mouse anti-GFP (1 500; Abcam), mouse anti-HA (1 100; Covance) and rat anti-HA (1 500; Roche); Mouse anti-Bam A7 (1 50), mouse anti-Hts 1B1 (1 20) and mouse anti-Sxl (1 10) were obtained from the Developmental Studies Hybridoma Bank. J. Knoblich [21] and P. Lasko [24] kindly provided the anti-Mei-P26 antibodies. Secondary antibodies were conjugated to Alexa568, Alexa488, FITC, Cy3 or Cy5 (1 500; Molecular Probes and Jackson ImmunoResearch).

### Immunoprecipitation from Ovaries

Immunoprecipitation experiments were conducted as previously described [23], starting with extracts generated from 100 pairs of wild-type ovaries or 200 pairs of *bam^▵86^* ovaries. Immunoblots were probed using anti-HA 3F-10 (Roche; 1 5,000), anti-GFP (1 2,000) and anti-Sxl (1 100). Secondary antibodies used included goat anti-rat-HRP, goat anti-rabbit-HRP and goat anti-mouse (Bio-RAD; 1 3,000).

Immunoprecipitation experiments followed by reverse transcription PCR (RT-PCR) were performed as previously described [23]. Different cycles (24–28) were performed and the resulting products were run on 1% agarose gels and visualized using EtBr. Primers listed below were used to detect *nanos* 3′UTR and *actin* 3′ UTR:

actin 3′UTR-forward: 5′-GACGAGCTCGAAGGATCGCTTGTCTGG

actin 3′UTR-reverse: 5′-GACACGCGTTTTTCATTTTTTGTAGTTC

nanos 3′UTR-forward: 5′- CTGACGCGTAGAGGGCGAATCCAGCTC

nanos 3′UTR-reverse: 5′-CAGGCGGCCGCGTATTACGATATTGTAAG.

Quantification of RT products (qRT-PCR) was conducted as previously described [23]. CHROMO4 Continus Fluorescence Detector from Bio-Rad (CH001234) was used to perform qPCR and Opticon Monitor 4 was used for analysis. qPCR was run at 95°C for 10 minutes and 95°C 15 sec, 60°C 1 min for 40 cycles. Each reaction contained 1 µl DNA from the RT product, 0.4 µl of each primer, 10 µl master mix (Power Sybr Green PCR Master Mix from Applied Biosystems) and 8.2 µl distilled water. Each IP sample was analyzed in triplicate. The primers of each gene used for qRT-PCR are listed here:

tub3′UTR-qPCR-F1: 5′-GGTAACCGTCGAAATCAG

tub3′UTR-qPCR-R1: 5′-CTATACGTGTCTTTGTGG

nos3′UTR-qPCR-F1: 5′CAACGAACGATCACTCAAATCC

nos3′UTR-qPCR-R1: 5′GCCACGACGATTGAACAAG.

### Superdex G-200 Gel Filtration

*w^1118^*; [*bam*P-Bam::HA]/CyO; [*bgcn*P-*bgcn*::GFP]/TM3 females were placed on wet yeast for two days and dissected in running buffer (50 mM Tris-HCI (pH 8.0), 100 mM NaCI). The total volume of ovaries used in this experiment was approximately 500 µl. These ovaries were extracted using 1 ml of running buffer, spun at 14,000×g at 4°C for 10 min. The supernatant was applied to a Beckman TL-100 Ultracentrifuge and spun at 40,000 rpm for an addditional 30 minutes. The supernatant was filtered through a 0.22 µm filter and loaded onto the column.

The Superdex G-200 (Amersham) column was equilibrated with the running buffer. The column was run at 0.5 ml/min and 1.5 ml elutions were collected. Western blot analysis was used to assay for the presence of Mei-P26, Bam::HA, Bgcn::GFP, Sxl, Ter94 and Actin.

### Yeast Two-Hybrid

Entry clones (pENTR/D-TOPO vector) of *mei-P26*, *bam*, *bgcn*, *ago1* were generated using a pENTR Directional TOPO cloning kit (Invitrogen) and subcloned into gatewayized pEG202 or pJG4.5 destination vectors. To test interactions in yeast, standard LexA-based two-hybrid assays were used.

## Results

### Bam interacts with members of the Mei-P26/Ago1/Bgcn/Sxl complex

Several previous studies have linked the activities of *mei-P26* and *bam* [21], [22]. Bam itself interacts with Bgcn and recent studies suggest that Bam cooperates with Sxl to promote cyst development [14], [20]. Immunofluorescence shows the expression of Mei-P26 overlaps with a GFP-tagged Bgcn transgene driven by its endogenous promoter (Figure). Mei-P26 expression also overlaps with Sxl in the anterior of the germarium (Figure) and Bam in cystoblasts and 2-, 4- and 8-cell cysts (Figure).

**Figure 1:**
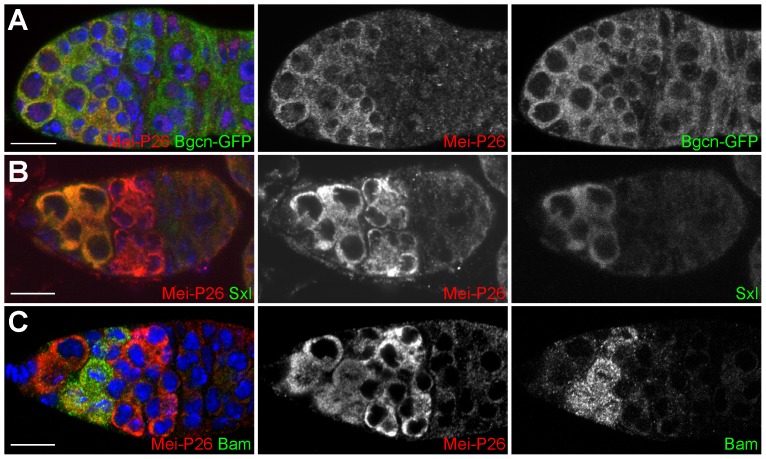
Germline cells within germaria co-express Mei-P26, Bgcn, Sxl and Bam. (A) A wild-type germarium stained for Mei-P26 (red), GFP-Bgcn (green) and DNA (blue). (B) A wild-type germarium stained for Mei-P26 (red), Sxl (green) and DNA (blue). (C) A wild-type germarium stained for Mei-P26 (red), Bam (green) and DNA (blue). Scale bars represent 10 µm.

Given their overlapping expression patterns and the genetic requirement for these factors during germline differentiation, we tested if Mei-P26 physically interacts with Bgcn, Sxl and Bam. We started by performing co-immunoprecipitation experiments using epitope tagged transgenes in S2 cells. These experiments showed that V5-tagged Mei-P26 associates with Myc-tagged Bgcn (Figure). Given the male embryonic origin of S2 cells, this interaction is likely independent of Sxl or Bam. Next, we tested whether endogenous Mei-P26 physically interacts with Bgcn, using the same GFP-tagged Bgcn transgene shown in Figure. These experiments indicated that Mei-P26 binds to Bgcn in both whole ovary extracts (Figure) and in *bam* mutant extracts (Figure), which are enriched for material from germ cells locked in a pre-cystoblast state. Finally, we used yeast 2-hybrid analysis to test for interactions between Mei-P26, Bgcn, Bam and Ago1 (Figure). Previous studies showed direct interactions between Bam and Bgcn using GST-pull down assays and co-immunoprecipitation of Mei-P26 with Ago1 [13], [21], [23]. In the yeast 2-hybrid experiments presented here, Bam and Bgcn associated with one another, as predicted. In contrast, Ago1 did not interact with Mei-P26, suggesting that other proteins may be involved in bridging the interactions between these proteins. Interestingly, Bgcn and Mei-P26 bait and prey constructs interacted in this yeast 2-hybrid assay, suggesting that possibility that these two proteins can associate directly with each other (Figure).

**Figure 2:**
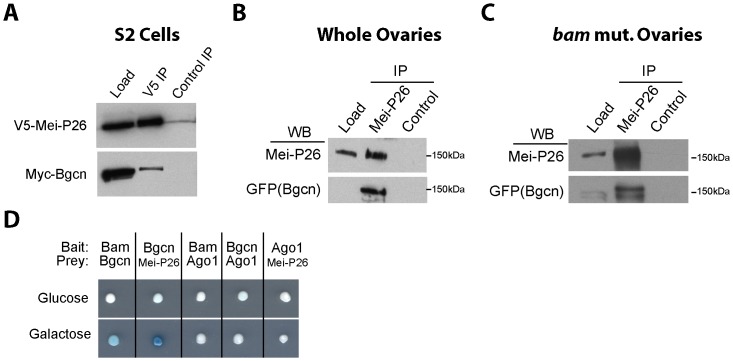
Mei-P26 physically interacts with Bgcn. (A) V5-tagged Mei-P26 co-immunoprecipitates Myc tagged Bgcn from S2 cell extracts. GFP-Bgcn immunoprecipitates with Mei-P26 in (B) wild-type whole ovary extracts and (C) *bam^▵86^* mutant ovary extracts. (D) Various combinations of Bam, Bgcn, Mei-P26, Ago1 bait and prey constructs were tested in a LexA based yeast-2-hybrid β-galactosidase assay.

Next, co-immunoprecipitation experiments using whole ovary extracts suggested that Mei-P26 associates with Sxl (Figure). We sought to determine whether Mei-P26 also physically associates with Bam. While Bam is both necessary and sufficient for germ cell differentiation, developing cysts express only low levels of Bam protein, making the protein difficult to detect by western blot analysis. To circumvent this problem, we over-expressed a tagged *bam* transgene using a heat-shock inducible promoter in ovaries and then performed immunoprecipitations from the resulting extracts using anti-Mei-P26 antibodies. Using this approach we readily detected an association between Bam protein and Mei-P26 (Figure). Moreover, the incorporation of Bam into the complex did not appear to alter the ability of Mei-P26 to interact with Sxl (Figure).

**Figure 3:**
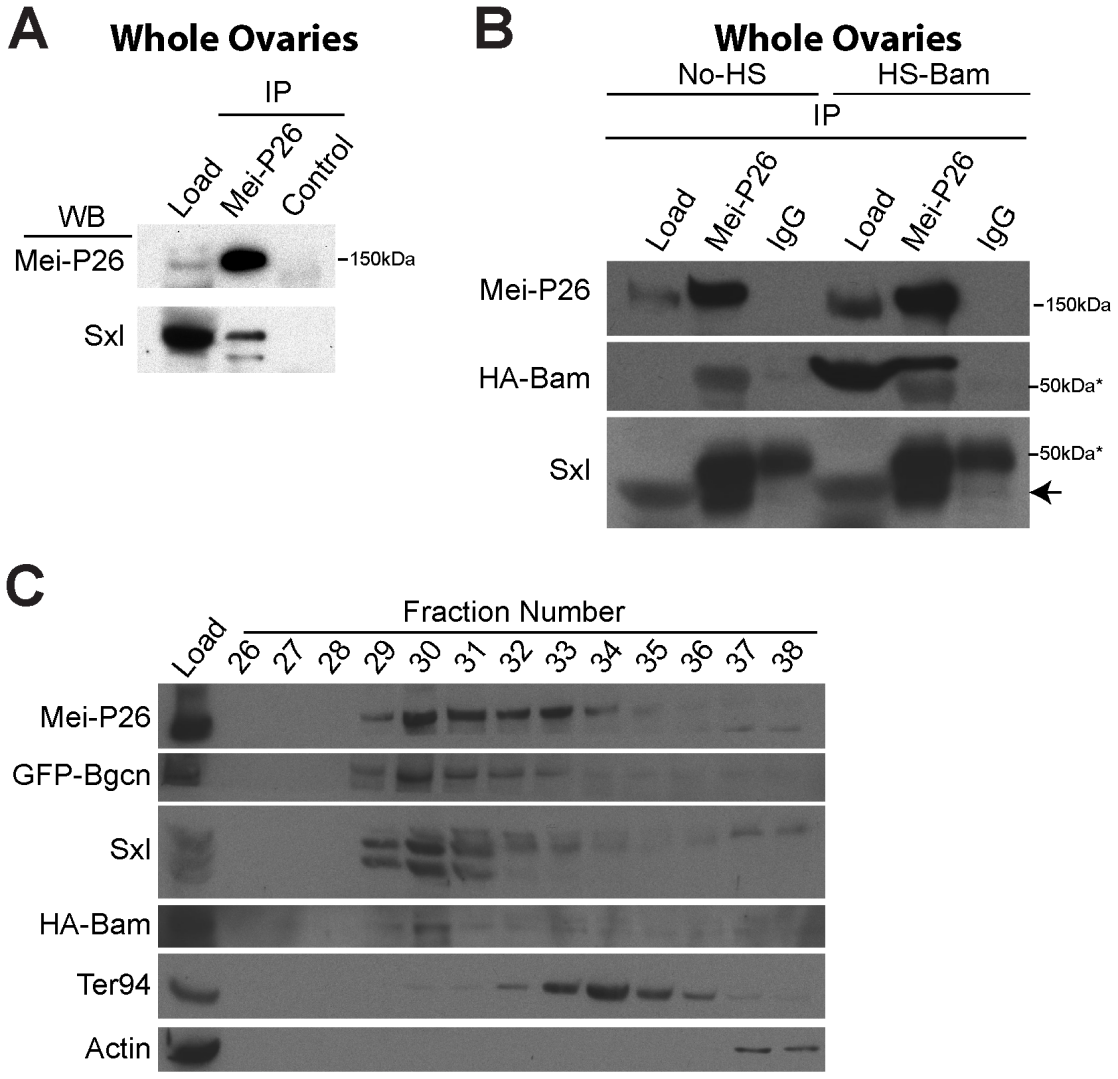
Sxl and Bam associate with Mei-P26. (A) Sxl immunoprecipitates with Mei-P26 in ovarian extracts from newly eclosed females. (B) Heat-shock induced Bam::HA and Sxl co-immunoprecipitate with Mei-P26. The levels of Sxl that associate with Mei-P26 do not appear to change in the presence of Bam::HA. (C) Extracts from whole ovaries were fractionated and the resulting eluents were probed for the presence of various proteins using western blot analysis. Mei-P26, GFP-tagged Bgcn, Sxl and HA-Bam were observed over a range of fractions but co-peaked in fraction 30. The control proteins Ter94 and Actin peaked in fractions 34 and 37 respectively.

If these four proteins formed a common, stable complex in vivo, they would be expected to co-fractionate from ovarian extracts. We prepared such extracts from flies expressing GFP-tagged Bgcn and HA-tagged Bam and performed size-exclusion chromatography. We then probed the resulting fractions for the presence of various proteins. Mei-P26, Bgcn, Bam and Sxl all co-fractionate, in a peak at fraction 30 (approximately 730 KDa; Figure). These data, together our co-immunprecipitation experiments, support the model that Mei-P26, Bgcn, Sxl and Bam physically associate with one another in the germline.

### Mei-P26 and Sxl cooperate with Bam-Bgcn to repress *nanos* translation

Previous data suggests that Bam, through either a direct or indirect mechanism, negatively regulates *nanos* translation during cyst development [13]. This repression results in a mutually exclusive expression pattern of Nanos and Bam in wild-type germaria (Figure; [13]) and is expected to be a key element driving cystoblast differentiation. To determine whether Mei-P26 helps to repress *nanos* translation as does Sxl [20], we compared the expression of these proteins in mutant ovaries. Salz and colleagues have circumvented some of the genetic complexity of eliminating Sxl from germ cells by using *snf* allele-specific properties [14], [25]. Consistent with previous reports [20], we found that *snf^148^* mutants exhibited overlapping Nanos and Bam protein expression (Figure). Similarly, *mei-P26^mfs1^* mutant germaria also displayed overlapping Nanos and Bam expression (Figure) and *mei-P26* mutant clones did not exhibit differences in the levels of Bam expression when compared to neighboring heterozygous germ cells (Figure). These results indicate that Mei-P26 participates in the repression of *nanos* translation in Bam expressing cells.

**Figure 4:**
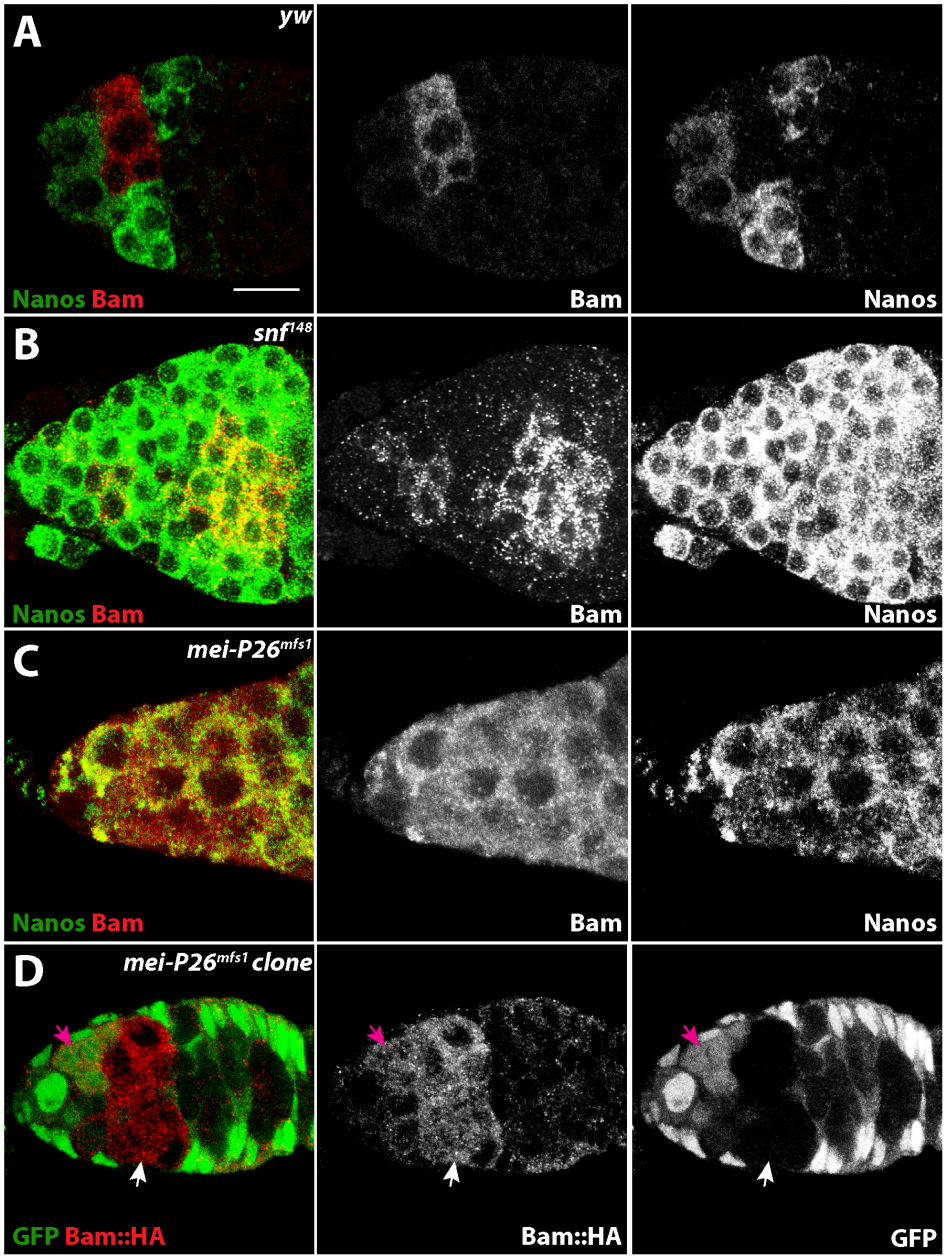
Loss of *snf* and *Mei-P26* results in mis-regulation of Nanos expression during cyst development. (A) Control, (B) *snf^148^* homozygous and (C) *mei-P26^mfs1^* homozygous germaria stained for Nanos (green), Bam (red) and DNA (blue). Overlapping Nanos and Bam expression is readily observed in both *mei-P26* and *snf* mutant ovaries. (D) A *mei-P26^mfs1^* clonal germarium stained for GFP (green) and Bam::HA (red). Both heterozygous cells (magenta arrow) and homozogous *mei-P26* mutant clones (white arrow) appear to express similar levels of Bam::HA. Scale bars represent 10 µm.

To determine whether Mei-P26 associates with *nanos* mRNA in cystoblasts and early cysts, we performed a series of immunoprecipitations and tested for the presence of *nanos* mRNA in the pellets using RT-PCR (Figure). We assayed for the presence of *actin* mRNA as a control for non-specific mRNA-protein interactions. As previously reported [20], we found *nanos* mRNA immunoprecipitated with Sxl (Figure). Furthermore, we detected *nanos* mRNA in Mei-P26 immunoprecipitation pellets as well (Figure). Both Sxl and Mei-P26 immunoprecipitations were repeated and the resulting pellets were subjected to quantitative reverse transcription PCR (qRT-PCR) (Figure). These additional experiments confirmed the initial RNA binding assays (Figure), and showed that Sxl and Mei-P26 consistently associated with *nanos* mRNA.

**Figure 5:**
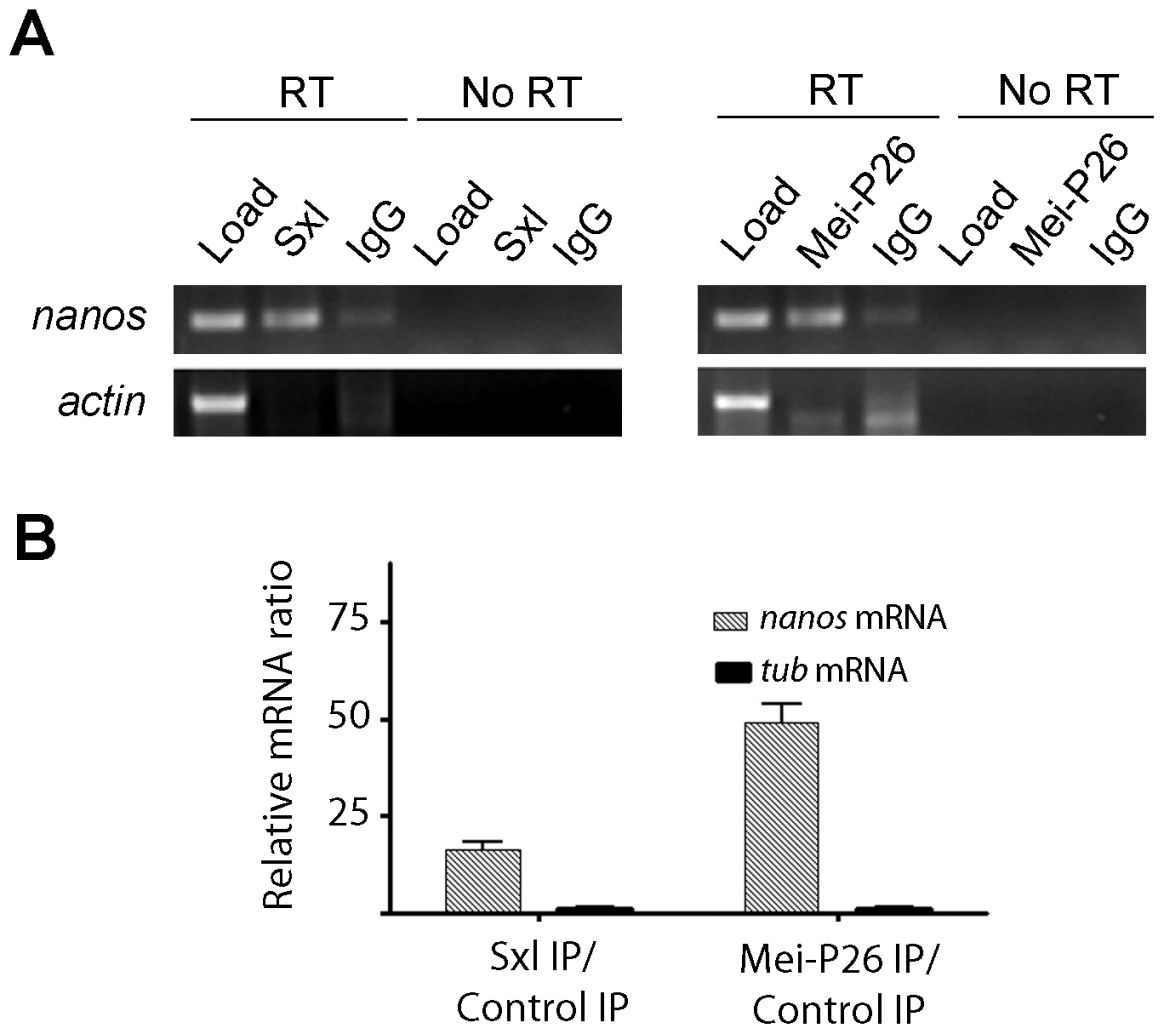
Mei-P26 and Sxl associate with *nanos* mRNA. (A) Immunoprecipitation of Sxl and Mei-P26 enriches for *nanos* mRNA but not *actin* mRNA. (B) Graph showing the ratio of mRNA pulled down in an Sxl IP versus a control IP and a Mei-P26 IP versus a control IP as measured by real time qualitative RT-PCR.

## Discussion

### Mei-P26 modulates translational repression in cystoblasts and early cysts

In this study we present data that Mei-P26 cooperates with Bam, Bgcn and Sxl to control the translation of *nanos* mRNA in the *Drosophila* female germline. Co-immunoprecipitation experiments indicate Mei-P26 physically associates with the differentiation factors Bam, Bgcn and Sxl and yeast 2-hybrid assays suggest the interaction between Mei-P26 and Bgcn may be direct. Disruption of *mei-P26,* or *snf*, which disrupts *sxl* expression in the germline, results in the upregulation of Nanos protein expression in early differentiating cysts. Both Mei-P26 and Sxl protein associate with *nanos* mRNA (this study; [20]). In light of the recently published study that shows mutating Sxl binding sites within the 3′UTR of *nanos* mRNA leads to mis-regulation of the gene [20], these results suggest that Mei-P26 may be part of a Sxl, Bgcn and Bam complex that serves to promote cyst development by directly repressing the expression of Nanos. However, despite repeated attempts, we have not been able to detect direct interactions between Bam and Bgcn with *nanos* mRNA. While various technical issues may prevent the detection of these specific interactions, our inability to observe direct association between Bam/Bgcn and *nanos* mRNA leaves open the possibility that interactions between the components of the Mei-P26, Sxl, Bam and Bgcn complex and its target mRNAs may be dynamic in nature. For instance, Bam and Bgcn may help to prepare Sxl and Mei-P26 for mRNA binding but do not themselves directly interact or only transiently interact with these targets. Further experiments will be needed to clarify the more specific molecular mechanisms that underlie Bam/Bgcn function with respect to the translational repression of *nanos* mRNA.

### Mei-P26 has multiple developmental functions in the germline

Two other recent studies investigated the role of *mei-P26* during germline development. Liu et al. (2009) showed that the RNA helicase Vasa directly regulates the translation of *mei-P26* mRNA through poly (U) elements within its 3′UTR [24]. Mutations in each gene strongly enhance the phenotype of the other, resulting in the formation of cystic germline tumors. Neumuller et al. (2008) focused on the function of Mei-P26, showing that it negatively regulates the activity of the miRNA pathway. We propose that Mei-P26 functions in both GSCs and early differentiating germ cells. Within GSCs, Mei-P26 is in a complex with miRISC proteins and enhances miRNA-mediated silencing [23]. In addition, Mei-P26 associates with Nanos protein and promotes BMP signaling within GSCs by repressing the expression of the negative regulator Brat [23], [26]. GSC daughters displaced away from the cap cell niche experience less BMP signaling, allowing for the expression of Bam.

We speculate that upon Bam expression, Mei-P26 switches its activity and/or its mRNA targets. This switch allows Mei-P26 to promote germline differentiation by both negatively regulating the miRNA pathway [21] and cooperating with Bam, Bgcn and Sxl to repress the translation of specific mRNAs such as *nanos* (this study). However the complex functional relationships between Mei-P26, Sxl, Bam and Bgcn remain incompletely understood. While we provide evidence that these factors can physically associate with each other under certain conditions, disruption of these genes results in two discrete phenotypes. *mei-P26* and *snf* mutants exhibit a cystic tumorous phenotype marked by the accumulation of undifferentiated cysts [14], [20], [21], [22] that do not express A2BP1, a molecular marker present in 4-, 8- and 16-cell cysts in wild-type samples [27]. In contrast, disruption of *bam* or *bgcn* results in the formation of single cell germ cell tumors [11], [12], [28]. These phenotypic differences suggest that Bam and Bgcn carry out additional functions independent of Mei-P26 and Sxl. A more complete characterization of the regulatory networks that govern the very early steps of germline cyst differentiation will have to await a better biochemical characterization of Bam and Bgcn function.

### The molecular function of Mei-P26

Together data presented here and elsewhere suggest that Mei-P26 has a variety of molecular functions inside and outside of the germline [21], [22], [23], [24], [29], [30], [31]. It remains unclear whether Mei-P26 exhibits the same biochemical activity when complexed with different proteins or whether its function completely changes depending on context. Based on the presence of a RING domain, Mei-P26 may act as an ubiquitin ligase. However this specific enzymatic activity has not been demonstrated nor have any direct in vivo substrates been identified. In regards to the translational repression of specific mRNAs, we favor a model in which Mei-P26 exhibits the same molecular activity within GSCs and their early differentiating daughters. We further speculate that association of Mei-P26 with different mRNA binding proteins modulates its targeting of specific mRNAs, and/or the degree to which these different targets are repressed. The expression of Bam correlates with changes in the development role of Mei-P26 but the manner in which Bam alters the composition or activity of the Mei-P26 complex remains unknown. Regardless, the findings that Bam can associate with Mei-P26 and Sxl provide further support for the hypothesis that Bam regulates the translation of specific mRNAs to promote the early steps of differentiation within the *Drosophila* female germline.
